# Vigilance, Resilience, and Intent to Pursue Medicine Among Underrepresented Students

**DOI:** 10.15694/mep.2021.000098.1

**Published:** 2021-04-26

**Authors:** Daniella Ortiz, Niki Matusko, Janice Vallie, Julie Burdine, Jonathan Finks, Gurjit Sandhu

**Affiliations:** 1 University of Michigan Medical School; 2 University of Michigan Department of Surgery

**Keywords:** Pipeline programs, Underrepresented in medicine students, Vigilance, Resilience

## Abstract

This article was migrated. The article was marked as recommended.

It is crucial that we understand what challenges still exist for underrepresented in medicine (URiM) students on the path to medicine in order to design more effective pipeline programs. Resilience and its relationship to success in medicine is a growing area of interest in medical education, and the concept of vigilance has been studied in the context of the health of racialized groups. We sought to measure the roles of resilience and vigilance on URiM students’ commitment to entering the medical field as well as the relationship between these two factors. A survey including the 10- item Connor Davidson Resilience Scale, the abbreviated Heightened Vigilance Scale, and questions measuring perceptions of everyday discrimination and intentions to pursue medicine was distributed to participants of Doctors of Tomorrow, a University of Michigan Medical School pipeline program focused on high school and undergraduate students. We detected significant relationship between resilience and intention to pursue medicine via Fisher’s exact test (p=0.004). There was no significant relationship between vigilance and intention to pursue medicine nor between vigilance and resilience. We conclude that including resilience development for URiM students in pipeline program curricula could enhance URiM student matriculation to medical school.

## Introduction

The crisis of underrepresentation in medicine (URiM) reflects a dearth of Black, Mexican-American, Native American, and mainland Puerto Rican students matriculating into and graduating from medical school. The ongoing gap perpetuating URiM limits the benefits inherent to working in diverse teams while also negatively affecting the care, treatment, and outcomes of minority patients ( Smedley *et al*., 2001; Institute of Medicine, 2003; Ely and Thomas, 2001; Stahl *et al*., 2009). While pipeline programs have been identified as one mechanism by which to address inequities facing URiM students, data still show that collectively medical schools are failing to reach representation of minority matriculants that reflects the demographics of the general population. For example, individuals who identified as Black and African American comprised 8.6% of medical school matriculants in 2018 while making up 13.4% of the overall US population( AAMC, 2019; U.S. Census Bureau, 2020). Studies focused on barriers and facilitators encountered by URiM students have informed the development of pipeline programs so that their curricula often incorporate outreach to high schools and middle schools, access to financial assistance, exposure to educational sessions, and mentorship to support minority students ( Miretzky *et al*., 2016; Bright *et al*., 2018; Vick *et al.,* 2018). However, limited successes with respect to increased URiM students indicate additional obstacles that are not currently being addressed.

One potential factor behind the limited success of pipeline programs is that they have not fully accounted for the everyday experiences of minority youth that they aim to recruit. Heightened vigilance has been identified as one of these everyday experiences affecting racialized groups ( Hicken *et al.,* 2013). The principles of vigilance that frame our discussion consist of the thoughts and behaviors underlying the anticipation and rumination stresses that minorities may have as they navigate environments that are predominantly white. For example, a black male student may have increased anticipation of being suspected of not belonging on campus if he leaves his dorm room without a backpack. That increased anticipation stress, or heightened vigilance, causes him to interact more carefully with his environment than his white peers. Vigilance is both a way of moving through daily interactions as well as a lens for understanding those events. Several studies have demonstrated a correlation with chronic vigilance and multiple physical and mental health measures including depressive symptoms, self-rated health, and chronic conditions among individuals identifying as African American or Black ( Clark, Benkert and Flack, 2006; Hicken *et al*., 2013; Kershaw *et al*., 2017; McCluney *et al.,* 2018; Mayne *et al.,* 2019). Given the impact of vigilance on the mental and physical health of minorities, it is possible that the stress of chronic vigilance in individual students may be a contributing factor to the low matriculation of URiM students into a profession where they know they will have to constantly navigate largely white spaces. We have found no studies investigating whether heightened vigilance is manifested by pre-medical minority students or whether vigilance has any impact on their ultimate decisions to enter medical school.

Another potential driving factor behind students’ intention to pursue medicine is resilience - how well one adapts to adversity and changing events ( Palmiter *et al*., 2020). Research has shown that burnout is a pervasive issue amongst both medical trainees and practicing physicians ( Epstein and Krasner, 2013; Ishak *et al*., 2013; Bird and Pincavage, 2016; Card, 2018; Winkel *et al*., 2018). Studies have demonstrated that higher levels of resilience may be protective against burnout ( Back *et al*., 2016; Hlubocky, Rose and Epstein, 2017). Although resiliency has not been well-studied in the pre-medical student population, it is possible that low resiliency may present itself similarly as a mechanism of burnout in this group leading to low numbers of students who remain in pipeline programs through application to medical school. Understanding the role of resiliency among pre-medical URiM students requires further study.

This study aims to explore the relationship of vigilance and resilience among URiM students in a pipeline program and their intention to pursue medicine. We hypothesize that chronic vigilance may be associated with lower intention to pursue medicine. Furthermore, we hypothesize that higher resilience would be associated with higher intention to pursue medicine. It is important that we understand how these factors affect students in the pipeline program in order to address barriers and develop relevant curricula.

## Methods

### Design

This study employed a cross-sectional, survey-based design administered through the Doctors of Tomorrow (DoT) program (Derek *et al.,* 2016; Haggins, Sandhu and Ross, 2017; Derek *et al.,* 2018). DoT is a pipeline program initiated in 2012 between the University of Michigan Medical School and Cass Technical High School in Detroit. DoT provides hands-on experiences and mentorship for students from 9 ^th^ grade into university. Data collection occurred from June 2019 to July 2019. This study was approved by the University of Michigan IRB

### Participants and Setting

203 students were affiliated with DoT from September 2012 to June 2019. An email was sent to all the students inviting them to complete the survey. The invitation described the purpose of the study, explained that participation was voluntary, and included a Qualtrics link to the survey instrument (Qualtrics, Provo, UT). No incentives were given to complete the survey.

### Survey Instrument

The measures collected in this study, included: vigilance, perception of everyday discrimination, resiliency, and intent to pursue a medical field. The complete survey contained 31 items. The survey includes the abbreviated Heightened Vigilance Scale ( Hicken, Hedwig and Hing, 2017), the 10-item Connor Davidson Resilience Scale (CD-RISC) to evaluate resilience ( Conner and Davidson, 2003), an abbreviated version of the Everyday Discrimination Scale ( Williams *et al*., 1997), and an additional question measuring intentions to pursue medicine.

The abbreviated Heightened Vigilance Scale (see Appendix 1) is based on the work done by scholars in the area of race discrimination. Clark, Benkert and Flack conducted psychometric analyses on an adolescent sample and found that one component emerged from the scale and as such sufficiently measured the latent construct of vigilance among adolescents while exhibiting moderately high internal consistency (2006).

The 10-item Connor Davidson Scale (see Appendix 2) was used to evaluate resilience ( Connor and Davidson, 2003; Campbell-Sills, Ford and Stein, 2009). The 10 item CD-RISC is a subset of the original 25-item inventory. Campbell-Sills, Ford and Stein conducted a series of empirically driven modifications to the original 25-item scales which resulted in a 10-item unidimensional scale that demonstrated good internal consistency and construct validity (2009). Overall, the10-item CD-RISC displayed excellent psychometric properties and allowed for efficient measurement of resilience ( Davidson, 2018).

We used an abbreviated version of the Everyday Discrimination Scale (see Appendix 3) to measure perception of everyday discrimination ( Williams *et al*., 1997; Taylor, Kamarck and Shiffman., 2004; Krieger *et al.,* 2005).

To measure participants’ intentions to pursue a medical degree, we asked each participant to rank the statement “I plan to pursue a medical degree” on a 7 point agree to disagree score, where: 1= strongly agree, 2= agree, 3= somewhat agree, 4= neither agree or disagree, 5= somewhat agree, 6= disagree, 7= strongly agree.

The following demographic data was collected: age, race, gender, annual household income, highest maternal and paternal education, and individuals living in the home (see Table 1).

After pilot testing the questionnaire with a group of 5 DoT students, the order in which the items appeared in the survey was altered and free-text spaces were added so that respondents could include comments. The survey took approximately 5 minutes to complete. Participants could skip questions to which they did not want to provide a response. Respondents received two reminders via email to complete the survey and the link remained open for 2 weeks.

**Table 1.** Demographics of Respondents

Characteristics		n	%
Sex	Total	50	100
	Male	14	28
	Female	36	72
Age (years)	Total	50	100
	14	5	10
	15	8	16
	16	10	20
	17	12	24
	18	3	6
	19	4	8
	20	6	12
	21	2	4
Race	Total	54	100
	Black or African American	32	60
	Asian	10	19
	Latino	8	15
	White	3	6
Household Income	Total	42	100
	<$20,000	12	29
	$20,000-$34,999	4	10
	$35,000-$49,999	11	26
	$50,000-$74,999	9	21
	$75,000-$99,999	1	2
	>$100,000	5	12
Highest Maternal Education	Total	48	100
	Less than or some high school	14	29
	High school diploma or equivalent	6	13
	Trade, technical or vocational training or some college	3	6
	4-year college degree	15	31
	≥ 2 years graduate school	7	15
	I don’t know	3	6
Highest Paternal Education	Total	46	100
	Less than or some high school	8	17
	High school diploma or equivalent	14	30
	Trade, technical or vocational training or some college	3	6
	4-year college degree	14	30
	≥ 2 years graduate school	2	4
	I don’t know	4	9

## Results/Analysis

### Statistical Analysis

Factor analysis using maximum likelihood estimation (violation of normality was not severe) was used to assess the uni-dimensionality (all variables loaded on a single factor for each construct with loadings >0.70) of each construct (vigilance, resiliency, and everyday discrimination) and Cronbach’s alpha was used to assess internal consistency (alpha >0.7). The vigilance index was calculated as the count score of the four vigilance items and the resilience index was calculated as the count score of the 10 items. Likewise, the everyday discrimination scale was calculated as the count score of the seven discrimination variables. Lower count scores indicated lower levels of vigilance, resilience, and discrimination.

Continuous socio-demographic characteristics such as age, maternal education and paternal education are reported as means and standard deviations whereas categorical characteristics such as race, gender, and household income are reported as number of observations and percent. The primary outcome of interest, intent to pursue medicine, was dichotomized with the following coding structure: strongly agree/agree=1; all else not missing=0. The relationship between each socio-demographic variable and the outcome of interest was assessed via single factor logistic regression analysis. Independent t-tests were used to test for group differences between high levels of intent to pursue medicine and low levels of intent and vigilance, resilience, and everyday discrimination. Pearson’s product moment correlation was used to examine the relationship between vigilance and resilience and vigilance and everyday discrimination. Multivariable logistic regression analysis was used to assess whether or not significant relationships persisted while adjusting for demographic factors. All analyses were conducted in STATA15 and significance was set at p<0.05 ( Stata Corp, 2017).

### Results

Of the 203 students that were contacted, 59 participated in the survey as part of this pilot study. The majority of responses as reported in Table 1 were from 16-17 year olds (n=22; 44%). Most of the participants were female (n=36, 72%) and identified as Black or African American (n=32; 60%).

Most participants responded positively, with strongly agree or agree, to the item, “I plan to pursue a medical degree (n=41; 79%). There was no significant association between individual demographic factors and intentions to pursue a medical degree. There was also no significant association between individual demographic factors and levels of resilience.

Parent education level was associated with vigilance. There was a 2.67 (p=0.028) increase in vigilance (range: 4-16) for respondents whose parents have at least a college education or higher compared to those who have less than college. The other demographic factors were not associated with vigilance.

There was a statistically significant association between vigilance and everyday discrimination. For each unit increase in everyday discrimination, there was 0.40 (p<0.001) increase in vigilance while adjusting for gender and race. However, vigilance and resilience were weakly and negatively correlated (R=-0.19). There were no statistically significant relationships between intention to pursue medicine and vigilance or between intention to pursue medicine and perception of everyday discrimination. On the other hand, the odds of pursuing medicine in the future were statistically significantly higher (1.23, p=0.017) for each unit increase in resilience while adjusting for race and gender.

## Discussion

This study focused on better understanding the relationship among vigilance and resilience with intention to pursue a medical career among URiM students in the DoT pipeline program. We found that higher resilience among URiM students in the pipeline correlated with increased intention to pursue medicine. We also found that vigilance levels had no significant impact on the students’ intention to pursue medicine. While a significant positive correlation was found between vigilance and everyday discrimination, we detected no significant relationship between vigilance and resilience. Overall, this study demonstrates the importance of attending to both systemic and intrinsic factors in the development of pre-medical pipeline programs.

### Vigilance and Everyday Discrimination

The significant positive correlation between vigilance and everyday discrimination was not surprising in that it aligns with assumptions that as one experiences more episodes of discrimination, their anticipation for additional insults increases. Furthermore, given previous work showing the negative impacts of heightened vigilance on the physical and mental health of racialized groups ( Hicken *et al.,* 2013; Himmelstein *et al*., 2015; Hicken, Hedwig and Hing, 2017; Mayne *et al.,* 2019), we expected vigilance and a heightened sense of everyday discrimination to have a negative influence on pre-medical students’ capacity for resilience and on their intentions to pursue medicine. However, we did not find that increased consciousness of one’s minority racial status as it relates to health professions and the potential increased stress of navigating this space de-incentivized these students from pursuing a medical career. One possible explanation for this finding is that students are not currently perceiving medicine as a racialized space and instead perceive it to be an unknown space. Alternatively, URiM students may also have developed an awareness and resolve about their ability to navigate everyday discrimination with vigilance even in health professions; as such, there may be other gatekeeping mechanisms that continue to filter these youth out of medical school.

The one demographic factor that was significantly associated with vigilance was parental education. Students whose parents had higher education levels (college education or higher) reported higher vigilance levels than those whose parents had lower education levels. It could be assumed that those with exposure to advanced education would be more comfortable in a white dominated environment and, therefore, have less vigilance. However, previous surveys have shown that racial minorities with higher education levels are actually more aware of and report higher levels of discrimination ( Anderson, 2016; NPR, 2017). This may be due to the fact that increasing education levels lead racial minorities into increasingly white spaces and more opportunities to encounter barriers or prejudice based on one’s race ( Lewis and Henricks, 2017). It is logical that as experiences of discrimination increase, vigilance, or the anticipation of these insults, also increases. This relationship may be the reason that we see individuals with exposure to higher education displaying higher levels of vigilance in our student population.

We did not see a significant impact of vigilance on resilience levels. This finding is encouraging as it suggests that any increased vigilance in this student population is not a hindrance on their capacity for resilience. However, it is also possible that factors such as the design of our survey and the small population size of this study are contributing to this finding. For a more in-depth understanding of the null findings between vigilance and intention to pursue medicine and vigilance and resiliency, qualitative inquiry is needed.

### Resilience

Previous studies had shown the protective nature of resilience in medical training by demonstrating that higher levels of adaptability and superior coping skills seem to be protective against burnout ( Dunn, Iglewicz and Moutier, 2008; Taku, 2014; Winkel *et al*., 2018). Our findings indicate that as resilience levels among high school and undergraduate URiM students increase, the more they intend to pursue medical school. This finding is consistent with related studies indicating that resiliency as a protective trait could be fostered earlier in the pipeline trajectory to bolster their commitment to medicine. This was an encouraging discovery because it opens the possibility for immediate actionable changes. Resiliency training has already been developed and used with medical students. Elements of resiliency-building curricula include facilitated small groups strengthening practical wellness and coping strategies ( Aiello *et al*., 2011; Delaney, 2018), workshops on goal setting and expectation management ( Bird and Pincavage, 2016), teaching thought-reframing techniques ( Nedrow, Steckler and Hardman 2013), and individualized skill building through coaching ( Back *et al*., 2016). Given that resiliency programming already exists for medical students, it is possible that similar approaches could be adapted for pipeline program curricula which may play a role in increasing URiM students’ intention to pursue medicine.

There are limitations to this research. First, this study is based on findings from a single URiM program in the mid-West and may not be generalizable. Nonetheless, they provide evidence as a pilot study to further explore intrapersonal factors among URiM individuals interesting in pursuing medical careers. Second, individuals volunteered to participate in this study; therefore, there is the possibility of self-selection bias. Finally, the small sample size of participants was a limiting element for extensive analysis. A larger dataset including more students from multiple pipeline programs is necessary to substantiate these findings.

## Conclusion

In summary, our findings suggest that resilience may be a key factor in sustaining the intention to pursue medicine amongst URiM high school and undergraduate students. More research is required to understand how to best incorporate resilience training into current pipeline program curricula in order to capitalize on the benefit of increased resilience levels for URiM pre-medical students. While we did not find that vigilance or perceptions of everyday discrimination have an impact on URiM students’ intention to pursue medicine, further qualitative research is needed to fully investigate the everyday experiences of these students that may be impacting their ability to sustain their pursuit of a career in medicine.

## Take Home Messages

• Pipelines for URiM students continue to leak
• Everyday experiences of URiM students are possibly being overlooked
• High vigilance does not preclude URiM students from pursuing medicine
• Resilience level is a significant factor in commitment to medicine

## Notes On Contributors

**Daniella Ortiz** is a 3rd year medical student at the University of Michigan. She earned her B.S. in Neuroscience from the University of Pittsburgh. ORCID ID: https://orcid.org/0000-0002-9159-972X

**Niki Matusko**, MS is a senior statistician and currently provides statistical consultation, interpretation, and analysis to surgery residents across the Department of Surgery and their faculty mentors at the University of Michigan.

**Janice Vallie** is an Administrative Assistant working for the Chairman’s Office and the Education Research Sciences Collaborative at the University of Michigan. She earned her B.S. from Eastern Michigan University in Sociology with a minor in Psychology.

**Julie Burdine** is a research specialist with a background in qualitative research in social and behavioral psychology. She received her B.A. from the University of Michigan and is currently completing her Master’s degree in health psychology. ORCID ID: https://orcid.org/0000-0001-8974-7597

**Jonathan F. Finks**, M.D, is a Assistant Professor in the Division of Minimally Invasive Surgery, Section of General Surgery. He joined the faculty at the University of Michigan Health System in August of 2005 and founded the Doctors of Tomorrow pipeline program in 2012.

**Dr. Gurjit Sandhu** is a faculty member in the Department of Surgery. Dr. Sandhu earned her PhD in 2006 from Queen’s University, Canada. Her work focuses on the scholarship of teaching and learning, specifically looking at professional education, teaching methods and assessment. ORCID ID: https://orcid.org/0000-0003-0258-7899

## Figures and Tables

**Table:** 

Try to prepare for possible insults from other people before leaving home.
Feel that you always have to be very careful about your appearance to get good service or avoid being harassed.
Carefully watch what you say and how you say it.
Try to avoid certain social situations and places.

**Table:** 

I am able to adapt when changes occur.
I can deal with whatever comes my way.
I try to see the humorous side of things when I am faced with problems.
Having to cope with stress can make me stronger.
I tend to bounce back after illness, injury, or other hardships.
I believe I can achieve my goals, even if there are obstacles.
Under pressure, I stay focused and think clearly.
I am not easily discouraged by failure.
I think of myself as a strong person when dealing with life’s challenges and difficulties.
I give my best effort no matter what the outcome may be.

**Table:** 

Are you treated with less courtesy or respect than other people?
Do you receive poorer service than others at restaurants or stores?
Do people act as if you are not smart?
Do people act as if they are afraid of you?
Are you threatened or harassed?
Are you followed around in stores?

**Table:** 

Your ancestry or national origin
Your gender
Your race
Your age
Your religion
Your height
Your weight
Some other aspect of your physical appearance
Your sexual orientation

## Appendices

**Appendix 1:** Abbreviated Heightened Vigilance Scale

In your day-to-day life, how often do you do the following things?

The answer scale is 5-point Likert scale where 1=at least once a week; 2=a few times a month; 3=a few times a year; 4=less than once a year; 5=never

**Appendix 2:** 10-item Connor Davidson Scale

For each item please indicate how much you agree with the following statements as they apply to you over the last month. If a particular situation has not occurred recently, answer according to how you think you would have felt.

The answer scale is 5-point Likert scale where 1=not true at all; 2=rarely true; 3=sometimes true; 4=often true; 5=true all the time

**Appendix 3**: Everyday Discrimination Scale

In your day-to-day life how often...

The answer scale is 5-point Likert scale where 1=at least once a week; 2=a few times a month; 3=a few times a year; 4=less than once a year; 5=never

Thinking about these experiences which have happened to you, overall what do you think was the main reason for these experiences:
