# Supplementary material for: Vigilance, Resilience, and Intent to Pursue Medicine Among Underrepresented Students
Source: MedEdPublish (2016). 2021 Apr 26;10:98. [Version 1] doi: 10.15694/mep.2021.000098.1 (PMC10939657; doi:10.15694/mep.2021.000098.1)
Supplement: Peer review history

# Reviewer report: Reviewer response for version 1

**DOI:** 10.21956/mep.18900.r27001

**Published:** 2021-10-09

**Authors:** Michelle McLean

**Affiliations:** 1 Bond University

This review has been migrated. The reviewer awarded 4 stars out of 5 Thanks for asking me to review this paper. I read it with interest and congratulate the first author (a student) on tackling this important issue. Taking into consideration that the numbers are small, which is duly acknowledged, the topic advances our understanding about some of the factors that may impact on historically marginalised student groups' To succeed in medicine, one certainly does need to be resilient. The concept of vigilance as a possible factor was a new line of research for me. To continue this work, a longitudinal study would be interesting. How many of these students go on to study medicine? Do these factors continue to be significant/not significant during medical studies? You may have to collaborate with other institutions so that your study has statistical power.

Reviewer Expertise:

NA

No decision status is available

# Reviewer report: Reviewer response for version 1

**DOI:** 10.21956/mep.18900.r27003

**Published:** 2021-10-04

**Authors:** Lisa Yarbrough

This review has been migrated. The reviewer awarded 3 stars out of 5 This paper is relevant to all administrators of URiM pipeline programs. Despite the emergence of pipeline programs, the number of underrepresented in medicine (URiM) matriculating into and graduating from medical school continues to not reflect the demographics of the U.S. population. Why is this so? While pipeline programs are only one mechanism to increase URiM enrollment, their curricula are informed by studies on external barriers and facilitators of URiM students. Your well-written introduction successfully sought a gap in the literature by shifting focus to the lived experiences of URiM students in a pipeline program, specifically heightened/chronic vigilance and resilience. Resilience and vigilance are additional factors that may need to be addressed by pipeline programs. Although the paper had a small sample size relative to the number of participants, the research methods were appropriate, well-conducted and reproducible. I recommend providing a definition of URiM in the introduction. Why were Asian and White participants included in the study? Also, in your table, the total n for Race is 52 and income is 42. There were 59 participants in total. Did the other participants not share the information? You noted a positive correlation between resilience and intent to pursue medicine. Your discussion links back to your stated hypotheses and does an excellent job exploring the results. Your study lends credence for vigilance, resilience and additional factors of the lived experience to be further examined/examined for inclusion into pipeline program curricula. While your findings are interesting, I recommend offering more specific ways the findings could be used.

Reviewer Expertise:

NA

No decision status is available

# Reviewer report: Reviewer response for version 1

**DOI:** 10.21956/mep.18900.r27002

**Published:** 2021-07-12

**Authors:** Trevor Gibbs

**Affiliations:** 1 AMEE

This review has been migrated. The reviewer awarded 4 stars out of 5 Although I had to read this paper through several times, I did find it interesting although slightly confusing; although I admit that may be due to my poor understanding of the subject. What came over very clearly to me however what the idea of vigilance which was made very clear in the paper.I could find very little to not suggest that this paper was of interest to all involved in student admissions and increasing student diversity; p a larger study is indicated to increase the credibility

Reviewer Expertise:

NA

No decision status is available
